# Use of Excimer Lamp and Topical Tofacitinib 2% Gel in the Treatment of Pediatric Alopecia Areata: A Case Report

**DOI:** 10.7759/cureus.83492

**Published:** 2025-05-05

**Authors:** Revathi Dineshkumar, Shvedha Manthri, Swetha Duraisamy, Abhijeet Malvi

**Affiliations:** 1 Dermatology, Revathi Hospital, Salem, IND; 2 Medical Affairs, Eris Lifesciences Limited, Ahmedabad, IND

**Keywords:** 308 nm excimer lamp, alopecia areata, pediatric, relapse, topical tofacitinib

## Abstract

Alopecia areata (AA) is a chronic inflammatory disease distinguished by non-scarring alopecia. The latest evidence suggests it is a T cell-mediated autoimmune disease that manifests in genetically susceptible individuals. In contrast to adult-onset AA, clinical traits and treatment of pediatric AA have been investigated very little. We hereby report a pediatric case of recalcitrant AA treated with a 308 nm excimer lamp and topical tofacitinib 2% gel.

## Introduction

Alopecia areata (AA) is a chronic inflammatory disease directed against the anagen hair follicles, causing non-scarring scalp alopecia and/or body hair. Pediatric cases accounted for 20% of AA cases [1]. First-line treatment with corticosteroids (topical or intralesional) is effective for many localized AA cases, but others may necessitate more aggressive approaches. In pediatric AA, there is a tendency for poor treatment response and pain intolerance with intralesional injections and the potential for more side effects with systemic treatment [1]. We hereby report a pediatric case of recalcitrant AA treated with a 308nm excimer lamp and topical tofacitinib 2% gel.

## Case presentation

**Figure 1:**
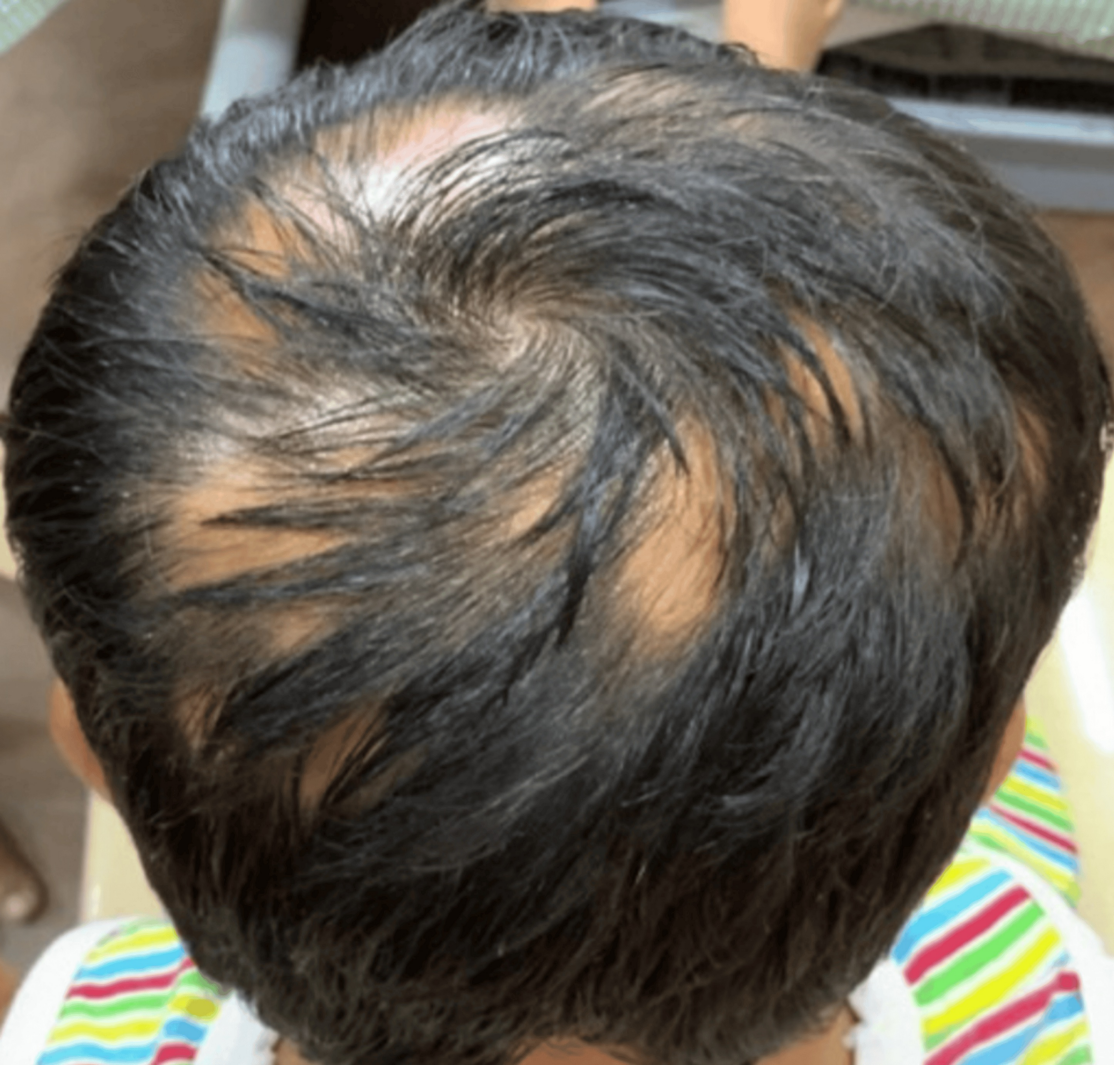
First visit to our clinic - SALT 23 (occipital view) SALT: Severity of Alopecia Tool

**Figure 2:**
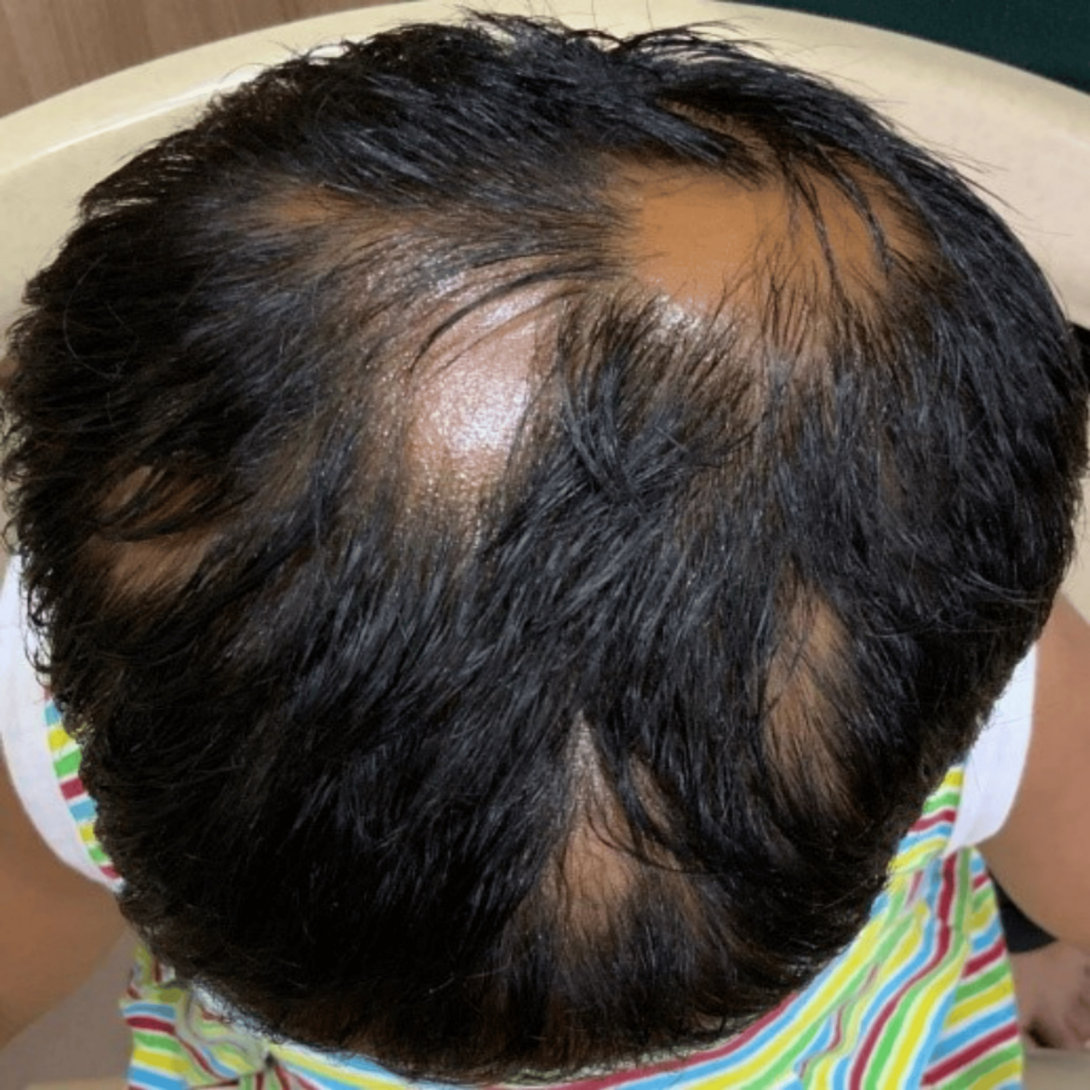
First visit to our clinic - SALT 23 (anteroposterior view) SALT: Severity of Alopecia Tool

A four-year-old female child weighing 16 kg was brought to the dermatology clinic by her mother with chief complaints of asymptomatic annular hair loss at multiple sites over the scalp for the past one year. The girl was having an O-positive blood group with completed vaccination to date and no history of atopy. No family history of similar conditions or allergic disorders was reported. On local examination, multiple well-defined annular patches of non-scaring alopecia of sizes ranging from 4 cm × 3 cm to 2 cm × 1 cm were present mainly at the vertex and parietal region of the scalp (Figures 1-2). Exclamatory hairs were present at the border, and the hair pull test was positive with no associated skin changes or nail changes. The Severity of Alopecia Tool (SALT) score before treatment was 23 (Table 1). The systemic examination was within normal limits. There were no abnormalities in the routine blood test.

**Table 1:** Excimer lamp (Dermaindia, Chennai, India): spot size specifications

Marking	Shape	Area (cm^2^)
D1	Circle	12.56
D2	Circle	7.06
D3	Circle	3.14
D4	Circle	0.78
D5	Square	6.25
D6	Square	1.00

The patient was treated with topical corticosteroids and weekend oral corticosteroid therapy for the past one year in an outside clinic and presented to our clinic with no significant improvement. Irradiation of the affected area was done with a 308 nm excimer lamp (Dermaindia, Chennai, India) once a week with a dose of 270 mJ/cm^2^ for 9 milliseconds with an appropriate spot size of 24 sessions for six months. Phototherapy with the 308 nm excimer lamp was added with the application of topical tofacitinib 2% gel once a day and oral multivitamins, calcium, vitamin D3, and iron supplements.

**Figure 3:**
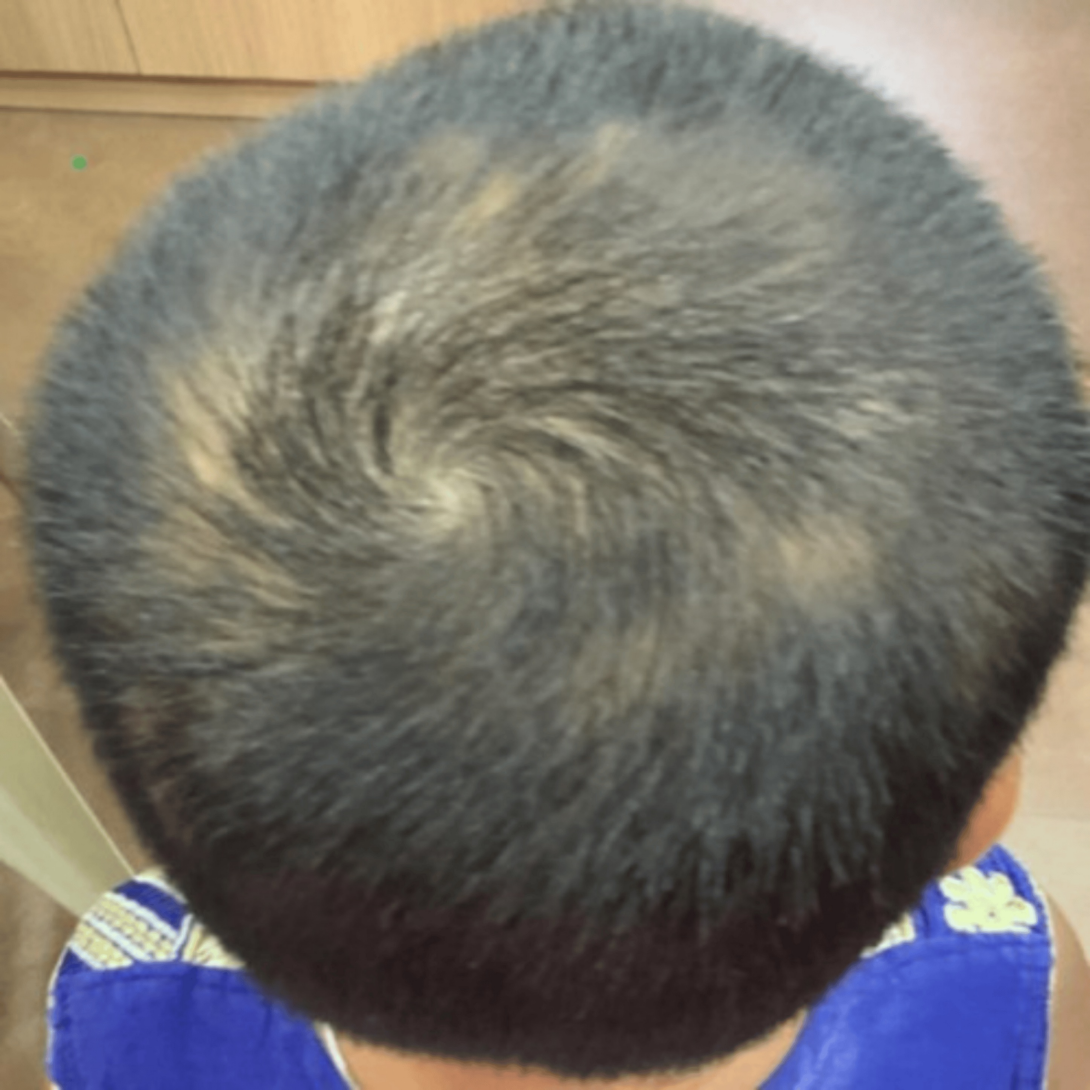
Fourth month of treatment (occipital view)

**Figure 4:**
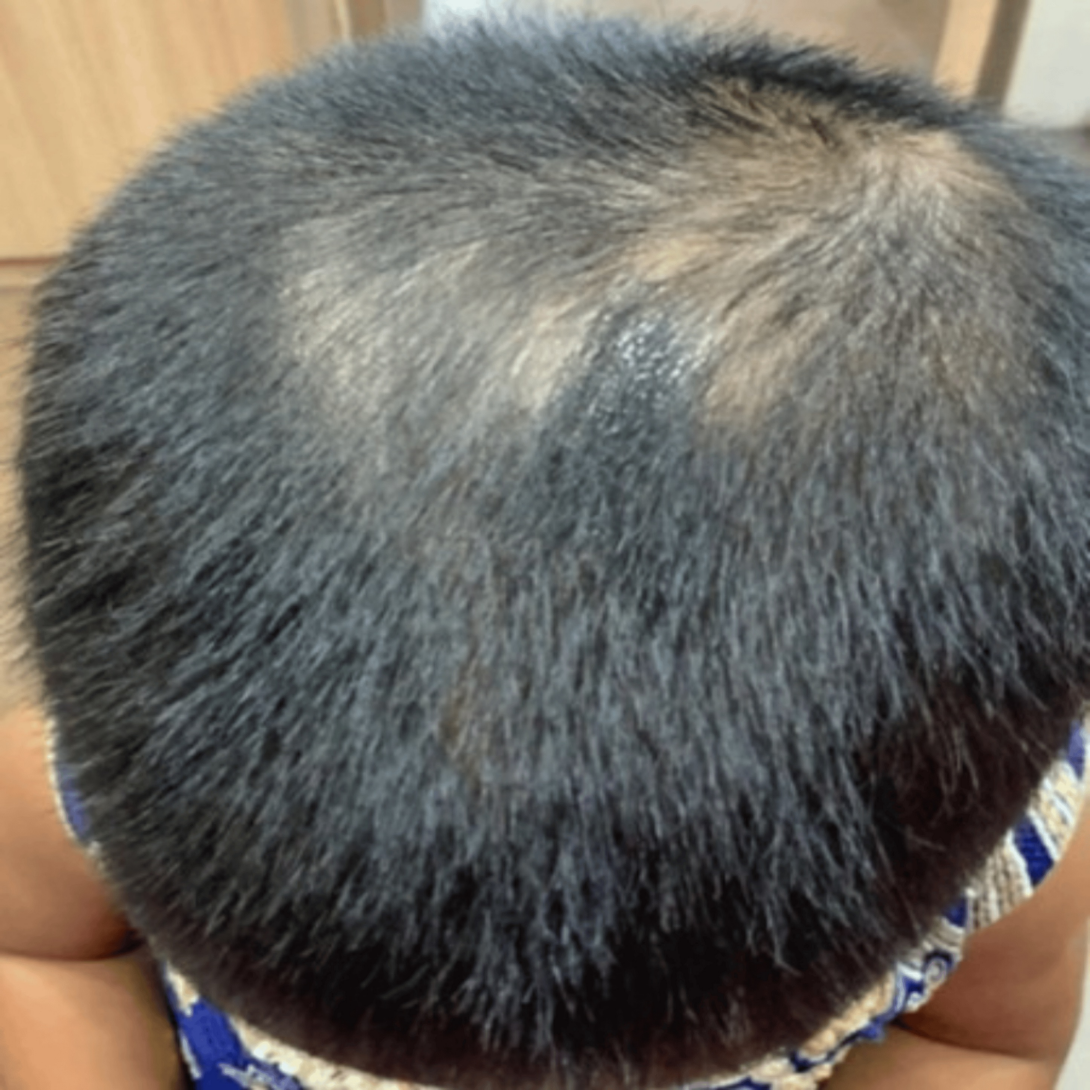
Fourth month of treatment (anteroposterior view)

**Figure 5:**
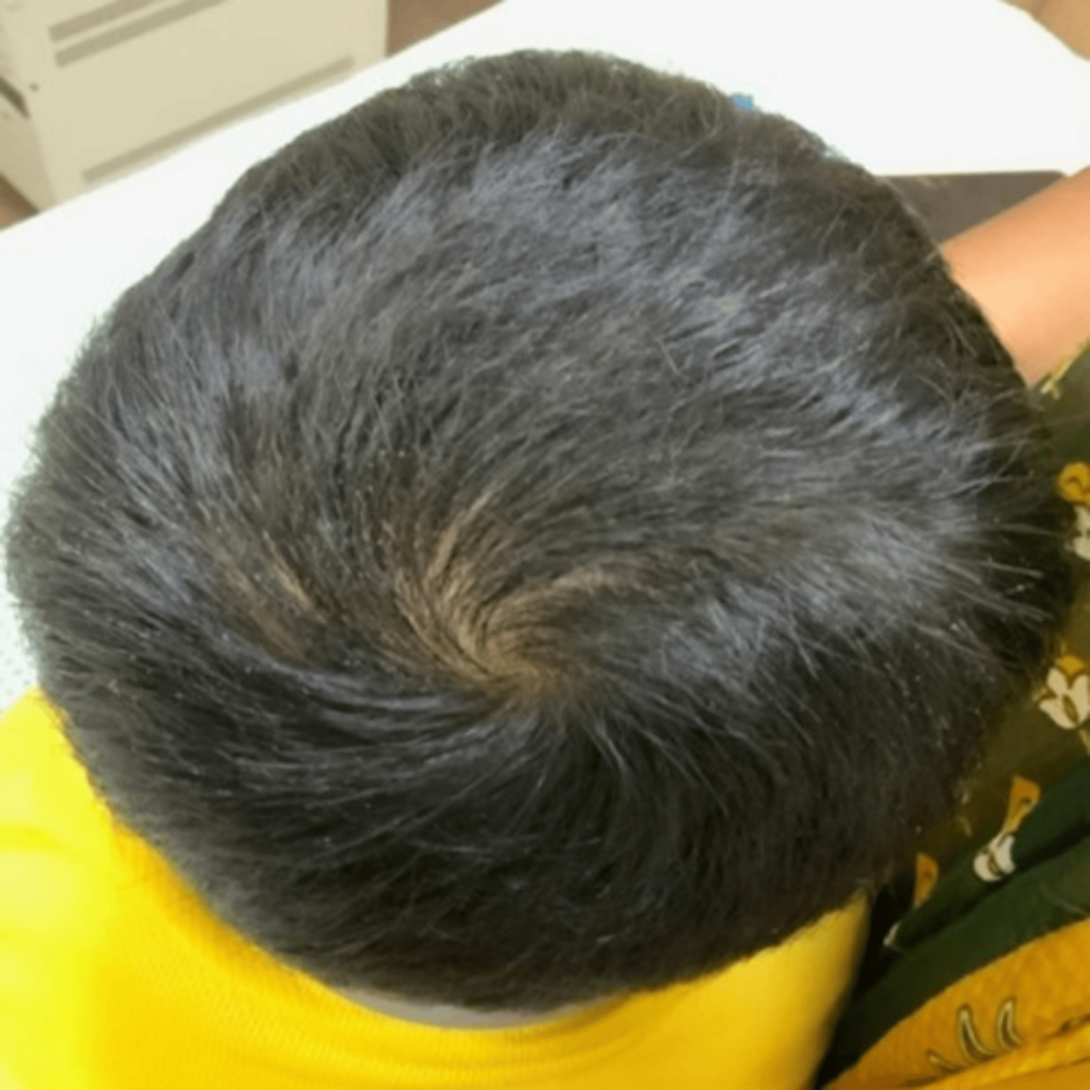
Sixth month of treatment

The patient had marked hair regrowth in four months (Figures 3-4) and complete hair growth in six months (Figure 5). No adverse effects were noted, and there was no relapse in subsequent follow-ups.

## Discussion

AA developing during childhood has a higher likelihood of extensive or refractory hair loss compared to adult-onset cases. Most conventional medical therapies available today have a low success rate and are frequently accompanied by a high risk of side effects [2].

The pathogenesis of AA involves autoreactive CD8+ T cell activation, primarily driven by JAK-STAT-dependent cytokines such as interferon-gamma and interleukin-15. Targeting interferon-gamma (IFN-γ) via JAK inhibition provides a rationale for JAKi use in AA management. Oral JAK inhibitors have demonstrated good effectiveness in managing refractory AA, but their long-term adverse effects are a matter of concern. Considering the systemic side effects of oral tofacitinib, the topical tofacitinib 2% gel is fairly well tolerated with no complications in pediatric AA [3]. As per a 24-week, open-label, single-arm pilot study involving 10 patients, hair regrowth was seen in three patients when treated with topical tofacitinib 2% ointment applied twice daily [4]. The mean reduction in SALT score was 34.6% (SD ±23.2%). One patient experienced significant scalp hair regrowth, with a 61% improvement in the SALT score, while two others had partial responses with SALT score improvements of 18% and 25%, respectively [4].

The 308 nm excimer lamp is safe and efficacious for managing AA. The photons of the excimer lamp act on the DNA of T cell lymphocytes and induce their apoptosis. Also, epidermal cellular DNA synthesis and mitosis are reduced. In addition to DNA injury in T cells, it also affects the relocation of T cells from the skin [5]. It enables targeted irradiation of the affected areas while preserving the surrounding unaffected skin [6,7]. Expert consensus recommends the simultaneous use of topical tofacitinib and light-based therapies such as psoralen and ultraviolet A (PUVA), narrowband ultraviolet B (NB-UVB), or excimer radiation to achieve superior results in managing vitiligo and AA [8].

Relapse or recurrence is an important aspect of the management of AA. Some of the factors linked to recurrence are earlier age of onset, family history of AA, a history of atopy, an autoimmune disease in patient or first-degree relatives, a disease duration of more than a year, associated nail changes, the appearance of new patch while on treatment, and severe nature of the disease, such as alopecia totalis, alopecia universalis, or emphasis pattern, more than 50% scaly involvement and loss of eyebrows and eyelashes. There were no adverse effects, and there was the absence of relapses on subsequent follow-ups [9].

## Conclusions

The case report demonstrated that for the pediatric patient with AA, phototherapy with a 308 nm excimer lamp and topical tofacitinib 2% gel is a valuable treatment option. It is also safe, effective, and painless, especially when dealing with the pediatric population. Moreover, the same treatment could be well tolerated and effectively reapplied in case of recurrence or relapse, without adverse effects.
